# Efficacy of entecavir and tenofovir in chronic hepatitis B under treatment in the public health system in southern Brazil

**DOI:** 10.1590/0074-02760150390

**Published:** 2016-04

**Authors:** Camila V Pereira, Cristiane Valle Tovo, Thiago K Grossmann, Henrique Mirenda, Bruna B Dal-Pupo, Paulo RL de Almeida, Angelo A de Mattos

**Affiliations:** Universidade Federal de Ciências da Saúde de Porto Alegre, Programa de Pós-Graduação em Hepatologia, Porto Alegre, RS, Brasil

**Keywords:** hepatitis B virus, therapy, nucleos(t)ide analogues, viral hepatitis

## Abstract

There are about 350 million hepatitis B virus (HBV) carriers worldwide and chronic HBV is considered a major public health problem. The objective of the present study was to assess the effectiveness of the nucleos(t)ide analogues tenofovir (TDF) and entecavir (ETV) in the treatment of chronic HBV. A cross-sectional study was carried out from March-December 2013, including all patients with chronic HBV, over 18 years of age, undergoing therapy through the public health system in southern Brazil. Only the data relating to the first treatments performed with TDF or ETV were considered. Retreatment, co-infection, transplanted or immunosuppressed patients were excluded. Six hundred and forty patients were evaluated, of which 336 (52.5%) received TDF and 165 (25.8%) ETV. The other 139 (21.7%) used various combinations of nucleos(t)ide analogues and were excluded. The negativation of viral load was observed in 87.3% and 78.8% and the negativation of hepatitis B e antigen was achieved in 79% and 72% of those treated with ETV or TDF, respectively. Negativation of hepatitis B surface antigen was not observed. There was no occurrence of adverse effects. This is a real-life study demonstrating that long-term treatment with ETV and TDF is both safe and effective.

About 40% of the world population present serological evidence of present or past infection by hepatitis B virus (HBV), corresponding to around 300-350 million HBV carriers worldwide (Hahné et al. 2013). Recently, the Ministry of Health of Brazil conducted a national survey in the country’s capitals to assess the prevalence of viral hepatitis, Brazil being considered an area of low endemicity for hepatitis B, with hepatitis B surface antigen (HBsAg) prevalence from 0.40-0.92% in different regions (MS 2010), although some areas are considered to be highly endemic (Souto et al. 1999). Thus, chronic hepatitis by HBV is still considered a public health issue, resulting in expressive morbidity and mortality rates around the world.

Two drug classes are available for the treatment of chronic infections by HBV: nucleos(t)ide analogues, which directly inhibit HBV-DNA replication, and interferon (IFN) alpha-based drugs, which can modulate the host response as well as viral replication. Nucleoside analogues [lamivudine (LAM), telbivudine, and entecavir (ETV)] and nucleotides [adefovir and tenofovir (TDF)] are currently available, as well as IFN-based drugs: conventional IFN (alpha 2a and 2b) and pegylated IFN (alpha 2a and 2b) (EASL 2012).

While international consensus establishes nucleos(t)ide analogues of high genetic barrier as firs-rate drugs in the treatment of HBV, the Brazilian public health system (MS 2009) prioritises TDF over ETV, probably for economic reasons.

There are no real-life studies in Brazil assessing the long-term response to nucleos(t)ide analogues as well as the occurrence of adverse events and the emergence of resistance in the treatment of patients with chronic hepatitis by HBV. Therefore, the evaluation of treatment response can pose practical applications for the population of patients treated in the public health system in Brazil.

The objective of the present study is to assess the effectiveness of the nucleos(t)ide analogues available through the public health system in Brazil (ETV and TDF) for the treatment of chronic hepatitis by HBV.

## PATIENTS, MATERIALS AND METHODS

This is a cross-sectional study conducted by reviewing medical records during the period from March-December 2013. All patients with chronic hepatitis B undergoing treatment through the public health system in southern Brazil, over 18 years of age, and presenting data for at least one reassessment of treatment in their medical records according to the rules established by the Brazilian public health system (MS 2009) were included.

Only data relating to the first treatment carried out with ETV or TDF in patients who had received no prior therapy was considered.

Patients who had undergone solid organ transplantation, co-infected with hepatitis C virus (HCV) and/or the human immunodeficiency virus (HIV), and subjected to immunosuppression for any reason were excluded from the study.

Available record data related to demographics (age, gender, ethnicity) and liver biopsy classified according to METAVIR score (Bedossa & Poynard 1996), as well as the type of nucleos(t)ide analogue used, usage time, and response to treatment were recorded. The assessment of response to treatment was performed using available data relating to viral load (VL) (quantitative HBV-DNA), hepatitis B e antigen (HBeAg), antibody to the e antigen (anti-HBe), HBsAg, antibody to the surface antigen (anti-HBs), and alanine aminotransferase (ALT) pre-treatment, at six months, at one year, and at the end of the monitoring period. The end of monitoring was defined as the last evaluation registered in the medical records for those patients who were under treatment for more than one year.

The response to the treatment was established by the occurrence of viral suppression, which was defined by the negativation of the VL.

The outcomes assessed in HBeAg-positive patients were ALT normalisation, HBeAg negativation, seroconversion to anti-HBe, negativation or reduction of HBV-DNA below the detection value, and HBsAg negativation with or without seroconversion to anti-HBs (EASL 2012).

For the HBeAg negative and anti-HBe positive (pre-core/core-promoter mutation) patients, the outcomes were ALT normalisation, HBV-DNA negativation or reduction below the detection value, and HBsAg negativation with or without seroconversion to anti-HBs (EASL 2012).

The emergence of virologic resistance was established when there was an increase of HBV-DNA (> 1 log) in the patients undergoing treatment after achieving virological response with previous HBV-DNA negativation (EASL 2012).

Assessment of renal function during treatment was performed evaluating serum creatinine.

Biochemical tests were performed in accordance with the recommendation of the Brazilian protocol (MS 2009). The HBsAg, HBeAg, and anti-HBe tests were performed using commercial radioimmunoassay tests in accordance with the manufacturer’s instructions. Quantitation of HBV-DNA was performed by polymerase chain reaction (PCR) (Saldanha et al. 2001), provided by the central public health laboratory.

The statistical package SPSS v.22.0 was used for the analysis of the results. Quantitative variables were presented as mean and standard deviation or mean and interquartile range when they were not normally distributed. For mean comparisons we used the Mann-Whitney *U* test. The qualitative variables were presented in the form of frequency and percentage. To check the associations between these variables, we used Pearson’s chi-square test with the additional feature of the adjusted residuals analysis to identify the location of the associations. The significance level was 5%. Regarding the calculated p-value, this was represented as < 0.001.

The data was obtained through the chart review in the public domain. The research project was approved by the Ethical Committee of the Federal University of Health Sciences of Porto Alegre (protocol 332-486/2013). The study followed the regulatory guidelines and standards for human research according to the resolution 466/2012 of the National Health Council.

## RESULTS

Six hundred and forty-eight patients were assessed. Of these, eight were excluded (2 for HIV co-infection, 2 for HCV co-infection, and 4 transplant recipients), totalling 640 patients. Regarding the choice of medication, TDF was used in 336 (52.5%) patients and ETV in 165 (25.8%) patients. Of the remaining patients, 61 (9.5%) used LAM and 78 (12.2) used various combinations of nucleos(t)ide analogues, being excluded from the present analysis.

Patients treated with ETV were significantly older than those treated with TDF. Caucasians represented more than 90% of patients in both treatments. Men were the majority, representing 80.5% in those treated with ETV and 64.6% in those treated with TDF (p < 0.001). The mean pre-treatment VL of patients using ETV was approximately three times higher than that of patients using TDF (p = 0.005). No statistically significant difference was found in the pre-treatment ALT values between patients with ETV/TDF.

Among the 165 patients treated with ETV, 135 underwent testing for HBeAg and, of these, 24 (17.8%) were HBeAg-positive, while of the 336 patients treated with TDF, 303 underwent testing for HBeAg, and of these, 25 (8.2%) were HBeAg-positive (p = 0.006). There were more cirrhotic patients treated with ETV than with TDF: six (13.6%) and two (2.2%), respectively (Table I).

**TABLE I:** Pre-treatment baseline characteristics of patients

Variable	Entecavir (n = 165)	Tenofovir (n = 336)	p
Age (years) [mean (SD)]	55.8 (12.1)	47.7(11.2)	< 0.001
Caucasian [n (%)]	157 (96.3)	312 (93.4)	0.220
Male gender [n (%)]	133 (80.5)	217 (64.6)	< 0.001
Viral load (IU/mL) mean (p25, p75)^*a*^	82,850 (1,670; 5,584,400)	27,998 (5,240; 1,001,600)	0.005
Initial ALT (U/L) mean (p25; p75)^*a*^	47 (27; 106)	39 (22; 79)	0.230
HBeAg positive [n (%)]	24 (17.8)	25 (8.2)	0.006
Cirrhosis [n (%)]	6 (13.6)	2 (2.2)	0.016

*a*: p25 and p75 represent the first and third quartiles, respectively; ALT: alanine aminotransferase; HbeAg: hepatitis B e antigen; IU: international units; SD: standard deviation.

All patients were under treatment during the whole period of evaluation. However, the number of patients evaluated in each period was not the same. From the total of patients, there were 254 patients evaluated at the 6th month of treatment, 294 evaluated at the end of the first year of treatment, and 162 evaluated at the end of the monitoring period. Patients who used ETV had been undergoing treatment for longer than those using TDF. Treatment time with ETV varied between 3.1-43.6 months (25% treated for a period of 12.2 months, 50% for 18.3 months, and 75% for 25.4 months). With regard to TDF, the treatment time varied between 3.1-42.6 months (25% treated for a period of 10.1 months, 50% for 16.3 months, and 75% for 22.3 months) (p = 0.015).

The number of patients with undetectable VL in the three monitoring periods was similar between the two medications (Table II). At the end of six months, the percentage difference in patients with undetectable VL between the medications was only 0.7% (p = 0.912). After one year of treatment, this difference reached 1% (p = 0.803) and at the end of the monitoring period the difference between medications was 8.5% (p = 0.126).

**TABLE II:** Analysis of viral load (VL) according to the treatment period

Assessment period				

	6 months	1 year	End of monitoring > than 1 year	p
Undetectable VL [n/n (%)]				
ETV	58/92 (63)	91/105 (86.7)	55/63 (87.3)	< 0.001
TDF	101/162 (62.3)	162/189 (85.7)	78/99 (78.8)	< 0.001
VL (IU/mL)				
ETV	601,131 (n = 92)	3,800 (n = 105)	499 (n = 63)	0.001
TDF	722,873 (n = 162)	43,591 (n = 189)	2,458 (n = 99)	0.001

ETV: entecavir; IU: international units; TDF: tenofovir.

It was observed that the VL prior to treatment was negative in 10.1% of patients using ETV and 2.8% in the group of patients using TDF. Throughout the treatment, there was a progressive increase in the number of patients with negative VL, reaching 87.3% and 78.8% at the end of monitoring for ETV and TDF, respectively, which was statistically significant for both treatments (p < 0.001). There was also a progressive decrease in median VL in both treatments during the follow-up period. The data related to the VL can be seen in Table II.

The development of resistance was observed in three patients (3.03%) using TDF and in one (1.59%) using ETV (p > 0.05).

Regarding HBeAg negativation, it was observed that in the pre-treatment period there were 24 (17.8) and 25 (8.2%) HBeAg-positive patients treated with ETV and TDF, respectively. At the end of the monitoring period there were five (3.9%) HBeAg-positive patients in the ETV group and seven (2.5%) in the TDF group (p = 0.508). Among patients who became HBeAg-negative, only three (1.8%) who used ETV and one (0.03%) who used TDF presented seroconversion to anti-HBe.

There were no cases of HBsAg negativation during the assessed period.

The different outcomes according to the medication used can be observed in Table III.

**TABLE III:** Different outcomes according to the medication used

	ETV [n/n (%)]	TDF [n/n (%)]	p
HBV-DNA loss	146/165 (88.5)	240/288 (83.3)	0.376
HBeAg loss	19/24 (79)	18/25 (72)	0.508
Anti-HBe seroconvertion	3/19 (15.8)	1/18 (5.56)	0.604
ALT normalisation	36/72 (50)	68/128 (53.1)	0.671

ALT: alanine aminotransferase; anti-HBe: antibody to the e antigen; ETV: entecavir; HBeAg: hepatitis B e antigen; HBV: hepatitis B virus; TDF: tenofovir.

Among the 79 patients with elevated ALT treated with ETV, normalisation was observed in 45 (59.5%) and, among the 133 patients with elevated ALT treated with TDF, 67 (50.4%) presented normalisation (p = 0.198).

There were no records of liver decompensation in cirrhotic patients or the emergence of hepatocellular carcinoma (HCC) during the monitoring period. Likewise, there were no records of the occurrence of adverse effects such as loss of renal function.

## DISCUSSION

The goal of antiviral therapy in chronic hepatitis B patients is the suppression of HBV-DNA, the negativation of HBeAg, its seroconversion, and, at last, to obtain the negativation of HBsAg with seroconversion to anti-HBs. Secondary outcomes are also expected, such as the decrease in mortality (EASL 2012, Liaw 2013). It has been reported that long-term treatment with nucleos(t)ide analogues can slow - and even reverse - the progression of fibrosis (Liaw 2013, Van Bommel & Berg 2013).

The majority of studies conducted in real-life with treatment-naïve patients receiving ETV or TDF were performed in Europe or in Asia (Ono et al. 2012, Hahné et al. 2013, Lin & Kao 2013). To our knowledge, there is only one study conducted in South America that assessed treatment-naïve patients treated with ETV in real-life (Ridruejo et al. 2014) and there are no studies assessing the use of TDF or comparing two analogues in HBV patients.

In the present study, it was possible to demonstrate that treatment with ETV and TDF is safe and effective, with VL negativation occurring in the majority of patients with both medications (87.3% and 78.8%, respectively). Likewise, HBeAg negativation was achieved in the majority of cases - in 79% (19/24) of those treated with ETV and in 72% (18/25) of those treated with TDF. However, seroconversion to anti-HBe was obtained in a small number of patients and there was no negativation of HBsAg. The development of viral resistance occurred in a small number of cases in patients using ETV and TDF (1.59% and 3.03%, respectively).

Although the assessed population had a higher mean age in the group of patients using ETV than in the group using TDF (55.8 and 47.7 years), as well as a higher male gender presence, there was no significant difference regarding ethnicity. This is comparable to the literature, where real-life studies included patients treated with ETV with a mean age between 36-58 years (Liaw 2013, Seto et al. 2013, Van Bommel & Berg 2013, Buti 2014, Chen et al. 2014, Ridruejo et al. 2014,Tsai et al. 2014) and patients treated with TDF were between 36-55 years of age (Lee et al. 2014, Ozaras et al. 2014), as well as a male gender majority in those treated with ETV, between 59-85% of cases (Chang et al. 2006, Lai et al. 2006, Marcellin et al. 2008, Zoutendijk et al. 2011,Buti et al. 2012, Ono et al. 2012, Tsai et al. 2012, Fahrtash-Bahin et al. 2013,Lin et al. 2013, Liu et al. 2013, Luo et al. 2013, Wang et al. 2013, Ridruejo et al. 2014), as well as TDF, between 50-74% (Marcellin et al. 2008, Lin et al. 2013). With regard to ethnicity, some authors included, in their studies, Asian patients who used ETV (Chang et al. 2006, Lai et al. 2006, Ono et al. 2012, Tsai et al. 2012, Luo et al. 2013) or TDF (Ke et al. 2014).

The mean treatment time in the present study was similar to other studies (Lai et al. 2006, Ono et al. 2012, Tsai et al. 2014), thus being sufficient to establish the effectiveness and safety of the treatments employed.

The VL negativation was achieved in 87.3% and 78.8% of patients who received ETV and TDF, respectively, at the end of the monitoring period. It has been described that the response to nucleos(t)ide analogues with VL negativation can reach more than 90% in one-two years of treatment (EASL 2012). In real-life, studies have shown VL negativation rates ranging between 44-100% in those treated with ETV (Zoutendijk et al. 2011,Buti et al. 2012, Ono et al. 2012, Tsai et al. 2012, Fahrtash-Bahin et al. 2013,Lin et al. 2013, Liu et al. 2013, Luo et al. 2013, Wang et al. 2013, Ridruejo et al. 2014) and between 76-97% in those treated with TDF (Lin et al. 2013).

HBeAg negativation was observed in 79% and 72% for ETV and TDF, respectively. According to the literature review, these values vary from 0-99% for ETV (Chang et al. 2006, Lai et al. 2006, Zoutendijk et al. 2011, Buti et al. 2012, Ono et al. 2012, Tsai et al. 2012, Fahrtash-Bahin et al. 2013, Lin et al. 2013,Liu et al. 2013, Luo et al. 2013, Wang et al. 2013, Ridruejo 2014) and from 0-100% for TDF (Marcellin et al. 2008, Lin et al. 2013).

In the present study, no cases of HBsAg negativation or seroconversion to anti-HBs were observed. Similar data was also observed in other studies (Liu et al. 2013), presenting low rates, or absence of HBsAg negativation and HBsAg seroconversion. In a recent systematic review (Tenney et al. 2009), HBsAg negativation occurred in 0-14% for ETV and 0-13% for TDF.

Patients with advanced liver disease have some risk of decompensation during treatment (Petersen et al. 2012, Ridruejo 2014) and some authors (Pan et al. 2014, Yu & Kim 2014) report the development of HCC after HBV treatment, showing that even with viral suppression, patients should be monitored for this complication. There were no records of hepatic decompensation or the emergence of HCC during the monitoring period in the present study.

Ridruejo (2014) carried out a recently published systematic review in which Phase III studies were included for hepatitis B treatment, with studies using ETV (Chang et al. 2006, Lai et al. 2006, Zoutendijk et al. 2011, Buti et al. 2012, Ono et al. 2012, Tsai et al. 2012, Fahrtash-Bahin et al. 2013, Lin et al. 2013, Liu et al. 2013, Luo et al. 2013, Wang et al. 2013, Ridruejo et al. 2014) or TDF (Marcellin et al. 2008, Lin et al. 2013). The average monitoring time varied between 12-58 months, totalling 4,681 patients. The HBV-DNA negativation occurred in 44-100%, HBeAg seroconversion in 8-68%, and HBsAg negativation in 0.2-14%. The average monitoring time varied from 12-33 months, totalling 574 patients, with HBV-DNA negativation in 76-97%, HBeAg seroconversion in 5-36%, and HBsAg negativation in 0-13%.

As observed in the present study, there were no significant differences between the two treatments (ETV or TDF).

Ozaras et al. (2014) carried out comparisons between patients treated with TDF (121 patients) and ETV (130 patients). Participants were selected from 10 care centres and the average treatment time was 17.7 ± 10.3 months. Patients showed comparable rates of HBV-DNA negativation (66.7% and 74.3%), HBeAg negativation (9.2% and 34.6%), and anti-HBe development (22.7% and 25.9%), respectively. Both medications provided effective viral control, with few side effects.

Ke et al. (2014) recently published a systematic review and meta-analysis including seven studies that compared ETV and TDF. Despite the small sample sizes in the analysed studies, there were no differences between the two medications [relative risk (RR) 1.10, 95% confidence interval (CI) 0.91-1.33 and RR 1.07, 95% CI 0.99-1.17 for 24 and 48 weeks of treatment, respectively), concluding that TDF and ETV are similar in effectiveness and safety at 24 and 48 weeks of treatment.

In general, studies observed a low incidence of resistance and viral escape (below 1% for ETV and TDF), with low levels of side effects and low incidence of discontinuation due to drug intolerance (Marcellin et al. 2013, Ke et al. 2014, Ozaras et al. 2014). In this study, the development of resistance was observed in four patients. However, it is not possible to define the influencing factors for such occurrences, since the irregular supply of the medications or nonadherence to treatment may be involved, factors that were not measured in this study.

TDF presents renal excretion with reports of renal function alterations in the literature (Ke et al. 2014). On the other hand, ETV presents a risk of lactic acidosis development in patients with decompensated cirrhosis (Seto et al. 2013). In the present study, there were no records of the occurrence of side effects potentially related to the use of these drugs. However, we understand that this data might be underestimated, as this is a retrospective study.

In conclusion, the present study differed from randomised clinical trials, being a study in real-life conditions and that adds information about long-term treatment effectiveness as well as safety in clinical practice. It was shown that both medications (ETV and TDF) have a high rate of HBV VL negativation and an excellent safety profile.
